# Letter from Professor Martins

**Published:** 1840-03-28

**Authors:** 


To the Editors of the Medical Examiner.

### FOREIGN CORRESPONDENCE.

### LETTER FROM PROFESSOR MARTINS.

### No. IV.

*Spirit of the French Medical Journals, and their influence during the year* 1839.

PARIS, 21st February, 1840.

To the Editors of the Medical Examiner.

IN politics, journals reflect the various opinions which divide a people, and so it is the case with medicine. No sooner do a certain number of physicians espouse a doctrine, than they feel prompted to make it known where it is not understood, to explain it where it has been misinterpreted, to defend it where it is attacked, and so a journal is created. Thus, since Bichât, and, after him, Broussais, agitated so many novel and interesting questions, disturbing from their sleep of lethargy, the great body of physicians who were reposing upon the pillow of tradition, the number of medical journals has been increasing from year to year. Be it said, however, to their honor, no one can be considered as undertaken for a commercial speculation. The majority cover their expenses, but few enable the proprietors to realize any profits. They continue to appear because they are the organs of a number of physicians who support them by their industry and subscriptions. The readers of the MEDICAL EXAMINER have been put in possession of all the more important productions of these journals during the past year; it has occurred to me, however, that it might not be uninteresting to present them a general review of these journals, and an estimate of their operations, drawn up by one who is personally acquainted with the editors, and can see and determine upon the spot the degree of influence which they exert upon the progress of the medical sciences.

*La Revue Médicale*, the oldest of the medical periodicals in France, numbers twenty years of existence. It is now under the sole editorship of M. CAYOL, who was formerly assisted by MM. Bayle, Gibert, and Martinet. This journal is the advocate of the vitalism of Hippocrates, and the representative of the school of Montpelier, personified by Barthez. It is constantly directing the attention of the physician to the observation of the physiological symptoms of disease, of the transformation they undergo, of their crises, and of the vix medicatrix naturæ. It insists upon the study of the constitution of diseases, of the epidemics which prevail, whence are to be deduced the characters presented by diseases at any epoch or locality. It rejects the numerical system, or rather it admits its application only to questions of time, frequency and duration. It is fond of that kind of generalization in which an author draws the picture of a disease which gives it, as it were, an artistical physiognomy. La Revue is a useful journal: it is a timely counterpoise to the efforts of those who are attempting insensibly to lead us to look at nothing in a patient but the lesions of organs, and to forget the disturbance of functions; of this tendency, which it designates by the name of *anatomism*, it is a most persevering adversary. It is a warm defender of the doctrines of the spiritualists, which it is constantly opposing to the inroads of a materialism, the dangers of which it exaggerates. Many eminent men enrich la Revue with their contributions, and, what is not a little remarkable, three of them are among the physicians who have most assisted to advance the progress of pathological anatomy, viz.: Professors Cruveilhier, Cayol and Recamier. When, therefore, we see men so competent to judge, continually warning the student of medicine to beware of the exaggerations of pathological anatomy, we cannot be inattentive to their caution, nor refuse to accord tc symptoms, of whatever nature, physical or physiological, their real value, deduced from observations and experience. The principal memoirs published by the Revue Médicale in 1839, are the following. An account of an operation for the cure of an hydatid tumour of the ovary, by M. Recamier. An essay on the therapeutic action of emetics, or the influence of these remedies upon the progress of diseases, by M. F. Andry. Experimental researches upon the oxide of iron as an antidote for arsenious acid, by MM. Deville, Sandras, Nonat and Guibourt. On vital energy, pathologically considered, by Dr. Bland. Clinical gatherings, by M. Payan. Researches on acute glanders, by MM. Nonat and Boulay. On the employment of the sensitive and intellectual faculties considered as a cause and means of cure of idiopathic headach (migraine,) by M. Lagasquie. On the seat, nature, and treatment of chlorosis, by M. Jolly.

Next in order and importance in the list of journals, comes the *Archives de Médicine*, which was established in 1823. It is, singularly enough, the journal which is the most formidable opponent of the Revue Médicale. Its collaborators are all graduates of the school of Paris, educated in her hospitals, and, in theory and practice, her representatives after the most exclusive fashion. Before M. Louis applied the numerical system to the study of medicine, the Archives was the journal the articles of which made the nearest approach to that system, and heralded the way for its speedy and inevitable introduction in the science. The memoirs in the Archives were most elaborately worked out, rich in observations and autopsies, and meagre in deductions. Thus, it never became the journal of practical physicians, properly so called. The latter have no love for writers who disturb their cherished beliefs and scatter doubts over their minds. They detest scepticism, and prefer "jurare in verba magistri." Hence, they naturally prefer to throw themselves into the arms of a man whose name is authority. They follow his dicta, act as he acts, treat as he treats, and leave him the responsibility of his own acts and of theirs. That such is the conduct of a large number of men, we have no difficulty in understanding, for it is in keeping with two inherent propensities of human nature, idleness and imitativeness. Now, the greater part of the long memoirs published in the Archives are often destitute of other conclusion than to point out deficiencies and errors hitherto unnoticed in the vast field of medicine, or to illustrate some obscure point in diagnosis or pathological anatomy. They are sometimes even terminated by the expression of the author's wish that his labours may be continued, himself finding them neither long nor full enough to justify any deduction whatever. For about two years past, this journal has been the organ of the numerical system which it advocates, with all its consequences. Its value and influence have been increased in the opinions of all scientific physicians who are under no apprehensions about the disturbance of their belief, or the destruction of their illusions. As the true Christian anxiously inquires of himself each day if he be in the path of truth, so they fear only error, and pursue it every where without intermission, lest it should deprive them of the most precious consolation of the physician, the certainty of having been always useful. You will not, then, be surprised to know that the Archives de Médicine does not enjoy the popularity that it deserves, considering the number and importance of its papers. The following have appeared during the year 1839. Researches upon some points of the semeiology of affections of the heart, by M. Beau. On phlegmonous tumours of the iliac fossæ, by M. Grisolles. Thoughts on typhoid fever, by M. Valleix. Memoir on a form of encephalitis, hitherto unnoticed, by M. Durand-Fardel. On the employment of douches and cold affusions in the treatment of insanity, by M. Leuret. On a particular alteration of the bladder in certain calculous affections, by M. Bouchacourt. On the pneumonia of children, by M. Alfred Bequerel. Researches on the gravid uterus, and on utero-placental apoplexy, by M. Jacquennier. Memoir on the treatment of varices, by M. Bonnet. On ulcerations of the air passages, by M. J. B. Barth. Essay on the curability of pulmonary consumption, by M. Hogée. On a new mode of diagnosticating various morbid changes of the prostate gland, by M. A. Mercier. On certain encysted tumours of the neck, by Messrs. L. Fleury, and L. Marchesvaux. On the effects of acetate of lead in the treatment of aneurism of the aorta, according to the *lessons* of M. Dupuytren. On the essential anatomical characteristics of yellow fever, by M. Louis. On the typhus and typhoid fever of England, by M. Valleix. Researches on the nature of tuberculous matter, by M. C. Baron. An examination of the various circumstances which appear in the course of disease, to determine the curved form of the nails, by M. Vernois. Fresh observations on the subjects of angina and tracheotomy, by M. Gendron.

The two periodicals of which we have just spoken, are devoted exclusively to medicine and surgery. But by the labours of MM. Parent-Duchatelet, Benoistan de Chateauneuf, Darcet, and Villermé, had, so to say, been created a new science, that of hygiene. These gentlemen had, with unequal sagacity and perseverance, devoted themselves to the investigation of the influence of occupations, vices, habits, locality, nourishment, &c., upon the health, statistics they employed as the only means of arriving at the truth ; but they tempered it with a sound spirit of criticism and analysis, the only mode of obtaining incontestable results. Hence' the memoirs produced under such auspices are admirable, not only from the importance of the facts established, but as models of logic and reasoning. For my part, I do not hesitate to pronounce *The Annals of Hygiene and Legal Medicine*, the most philosophical journal that we possess in France. In fact, the problems which its editors have attempted to solve, are the most difficult in medicine, involving, as they do, an accurate estimate of the general causes which influence the health of a population. In making the history of the case of a single isolated individual, how great is the difficulty in determining, from the number of morbific influences to which he has been exposed, that which has caused the disease which has attacked him. In spite of the closest investigation, we are often unable to decide it. When a large number of cases are concerned, how much greater is the difficulty. Is it an easy thing, for instance, to decide what renders a particular occupation unhealthy ? A superficial observer will have no trouble in answering the questions, for he has a stock of prejudices ready made for each case of difficulty? He accounts for the diseases of the persons who work in common sewers, privies, slaughter-houses of horses, and rope walks, from the deleterious miasmata which arise from these places. In the case of public women, it is the abuse of coition; and so on in other instances. And how careful should we be in our deductions, when we are aware that all these popular beliefs are erroneous, and incapable of standing sound criticism! The tendency of the *Annals of Hygiene and Legal Medicine* is to destroy these prejudices so deeply rooted for ages in the minds both of physicians and the public at large. This journal does the same service to medical jurisprudence, in subjecting to severe criticism all the doubtful cases brought before the courts, and publishing accounts of medico-legal consultations held by physicians of the highest ability in this speciality. I hesitate not then to repeat that this journal is the most useful and instructive that we possess, under whatever point of view we consider it. An enumeration of the articles published in 1839, will, I hope, bring most readers to the same opinion : On the mortality and insanity consequent on a penitentiary life, by M. Christopher Moreau. On drunkenness among factory operatives, by M. Villermé. On forced abortion, by Dr. Olivier of Angers. On the employment of the microscope in legal medicine, by Dr. Bayard. On foundlings, by Benoistan de Chateauneuf. Description of the apparatus to prevent soap boilers from falling into their cauldrons, by M. Darcet. On poisoning with the salts of lead, by M. Orfila. New signs of death by hanging, by M. Devergie. On the effects of swallowing pins and needles, by Dr. Olivier of Angers. On the influence of civilization in the development of insanity, by M. Leuret.

